# Dataset generated for Dissection of mechanisms of Trypanothione Reductase and Tryparedoxin Peroxidase through dynamic network analysis and simulations in leishmaniasis

**DOI:** 10.1016/j.dib.2017.10.031

**Published:** 2017-10-19

**Authors:** Anurag Kumar, Bhaskar Saha, Shailza Singh

**Affiliations:** National Centre for Cell Science, NCCS Complex, Ganeshkhind, SP Pune University Campus, Pune 411007, India

**Keywords:** TryR, Trypanothione Reductase, Txnpx, Tryparedoxin Peroxidase, TryS, Trypanothione synthetase, T(SH)_2_, Trypanothione, ProSA, Protein Structure Analysis, SAVES, Structure Analysis and Verification Server, MIPS, Munich Information Centre for Protein sequence, BIND, Biomolecular Network Interaction Database, DIP, Database of Interacting Protein, GRID, General repository for Interaction Database, MINT, Molecular Interaction Database, KEGG, Kyoto Encyclopaedia of Genes and Genomes, Trypanothione Reductase, Tryparedoxin Peroxidase, *L.major*, Homology modeling, Molecular clock analysis, Network analysis

## Abstract

Leishmaniasis is the second largest parasitic killer disease caused by the protozoan parasite *Leishmania*, transmitted by the bite of sand flies. It's endemic in the eastern India with 165.4 million populations at risk with the current drug regimen. Three forms of leishmaniasis exist in which cutaneous is the most common form caused by *Leishmania major*. Trypanothione Reductase (TryR), a flavoprotein oxidoreductase, unique to thiol redox system, is considered as a potential target for chemotherapy for trypanosomatids infection. It is involved in the NADPH dependent reduction of Trypanothione disulphide to Trypanothione. Similarly, is Tryparedoxin Peroxidase (Txnpx), for detoxification of peroxides, an event pivotal for survival of *Leishmania* in two disparate biological environment. Fe-S plays a major role in regulating redox balance. To check for the closeness between human homologs of these proteins, we have carried the molecular clock analysis followed by molecular modeling of 3D structure of this protein, enabling us to design and test the novel drug like molecules. Molecular clock analysis suggests that human homologs of TryR i.e. Glutathione Reductase and Txnpx respectively are highly diverged in phylogenetic tree, thus, they serve as good candidates for chemotherapy of leishmaniasis. Furthermore, we have done the homology modeling of TryR using template of same protein from *Leishmania infantum* (PDB ID: 2JK6). This was done using Modeller 9.18 and the resultant models were validated. To inhibit this target, molecular docking was done with various screened inhibitors in which we found Taxifolin acts as common inhibitors for both TryR and Txnpx. We constructed the protein-protein interaction network for the proteins that are involved in the redox metabolism from various Interaction databases and the network was statistically analysed.

**Specifications Table**

**Table:** 

Subject area	*Biology*
More specific subject area	*Network biology*
Type of data	*Text file, figure*
How data was acquired	*Literature Survey*
Data format	*Filtered and analysed*
Experimental factors	*Computational tools*
Experimental features	*Modeller, AutoDock Vina,Mr. Bayes,Cytoscape*
Data source location	*Pune, India*
Data accessibility	*Data is within this article.*

**Value of the Data**

• Trypanothione metabolism is important for the survival of leishmania inside the host macrophage.
• INO1 and RPN5 have been found to have a functional interaction and they are known to play a vital role in redox metabolism homeostasis.
• Taxifolin acts as common inhibitors for both Trypanothione reductase (TryR) and Tryapredoxin peroxidase (Txnpx).

## 1 Data

### 1.1 Leishmaniasis

Leishmaniasis is caused by trypanosomatids protozoan parasites that belong to the genus *Leishmania* and it is one of the most neglected parasitic diseases that affect about 350 million people together with two million new cases yearly. Leishmaniasis is seen frequently in Southeast Asia, Africa, South America, including mostly Brazil and Mediterranean countries **(WHO 2010).** The prevalence of disease in India is in West Bengal, Bihar, Jharkhand and Uttar Pradesh. Leishmaniasis has three clinical forms seen in humans, which are cutaneous leishmaniasis (*L.major, L.tropica* and *L.mexicana*); visceral leishmaniasis (*L.donovani* and *L.infantum*) that is most fatal and mucocutaneous leishmaniasis (*L. braziliensis*). The disease is transmitted by infected female sandflies to the vertebrate hosts where the parasites multiply within their macrophages in their amastigote form. The available drugs for leishmaniasis are antimony-containing compounds such as meglumine antimoniate, sodium stibogluconate (Pentostam) and other drugs include amphotericin, ketoconazole, miltefosine, paromomycin, and pentamidine [1], [2].

### 1.2 Trypanothione Reductase and Tryparedoxin Peroxidase

A rational approach to the design of new anti-leishmanial has identified a number of aspects in leishmania which may be exploited. One such target is the intracellular di-thiol Trypanothione unique to all kinetoplastidae including leishmania species and the Trypanothione (or homotrypanothione) is the principal low-molecular-mass thiol in these parasites which appears to have subsumed many of the anti-oxidant and other protective functions to glutathione in other organisms including mammals [3]. The intracellular level of dihydrotrypanothione and hence the reducing environment is maintained by the NADPH-dependent flavoprotein-disulphide-oxidoreductase, Trypanothione Reductase [7]. Although structurally and mechanistically analogous to the mammalian enzyme glutathione reductase, it belongs to class I Pyridine nucleotide –disulphide oxidoreductase family. It is a dimer which consists of two identical subunits A and B, each of which is 492 amino acids in length [6], [7].

Tryparedoxin peroxidases (Txnpx) are the terminal enzymes in the trypanothione dependent detoxification system in trypanosomatids. Electron transfer occurs between the Trypanothione Reductase and Tryparedoxin mediated by trypanothione. It accepts an electron from the Tryparedoxin for the reduction of the hydrogen peroxide and peroxynitrite. Tryparedoxin Peroxidases involved in the trypanothione dependent efflux system are essential, for the parasite survival [1], [4], [5], [10], [13].

### 1.3 Redox metabolism in parasites

**Fig. 1:**
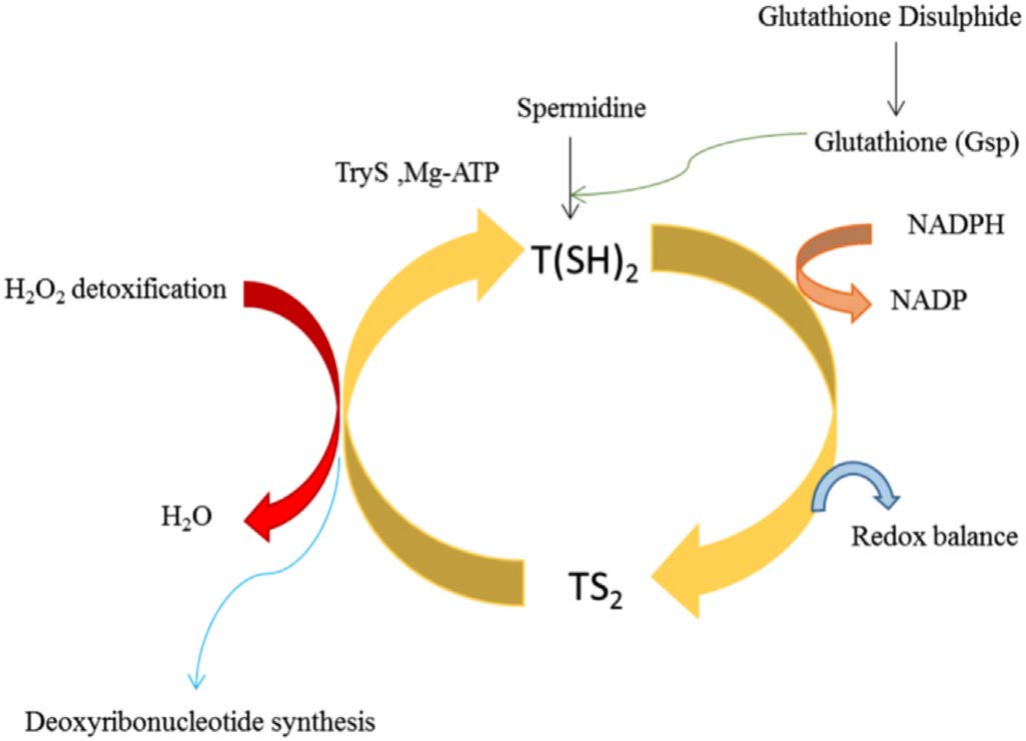
Redox Metabolism of *Leishmania*.

In mammalian host, the leishmania parasite proliferates in the macrophages. The parasite is able to survive in the host by the unique redox metabolism to fight against cells produced by host macrophages. Antioxidant mechanism of parasite relies on three enzymes namely Trypanothione synthetase (TryS), Trypanothione Reductase (TryR), Tryparedoxin Peroxidase (Txnpx) [2]. A dithiol Trypanothione [T (SH)_2_] is a central reductant metabolism of parasite redox pathway which is synthesized by TryS maintained in the reduced state by TryR enzyme. The [T(SH)_2_] transfers its reducing equivalent to thioredoxin and peroxidase that in turn reduces peroxidase for the detoxification of toxic reactive species generated during synthesis of hydro peroxide and deoxyribonucleotide. By keeping the Trypanothione in the reduced state the parasite facilitate the metabolic pathway (Fig. 1).

### 1.4 Fe-S clusters

Fe–S clusters constitute a ubiquitous class of protein prosthetic groups essential to life. Following the discovery of ferredoxins as Fe–S cluster containing proteins in the 1960s, the diverse chemical, electronic, and magnetic properties of Fe–S clusters were thoroughly characterized *in vitro*, and numerous synthetic routes for preparing Fe–S clusters of various chemical compositions have been documented [7]. The most common Fe–S cluster types are [2Fe–2S], [3Fe–4S], and [4Fe–4S] ligated to proteins through cysteine, histidine, or aspartate residues. Fe–S clusters confer unique functional properties to the proteins involved in vital cellular processes, including metabolism, photosynthesis, DNA replication and repair, and nitrogen fixation [9]. Fe–S clusters form spontaneously under anaerobic conditions *in vitro*; however, under oxidizing conditions *in vivo*, oxygen-labile. Fe–S clusters require intricate proteinaceous machinery for their efficient synthesis and transfer to apoproteins. Molecular oxygen rapidly degrades Fe–S clusters, and the resultant free iron ions, via Fenton chemistry, produce reactive oxygen species (ROS; e.g. hydroxyl radicals) that can inflict irreversible macromolecular damage, ultimately culminating in cell death. It participates in diverse biological processes such as respiration, central metabolism, DNA repair or gene regulation [14].

**Fig. 2:**
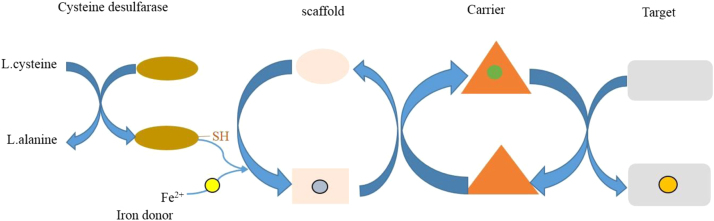
Fe-s cluster assembly.

Henceforth, it can be said that the Trypanothione metabolism is the most important metabolism for the survival of leishmania inside the host macrophage and, thus, it is essential to study the activity of TryR and Txnpx. As it is known, that those two proteins play a role in maintaining redox metabolism of leishmania, inhibition of these proteins may lead to anti-leishmanial effect that paves the way for designing the novel compound for leishmaniasis. Furthermore, the understanding of these proteins role in homeostasis of Fe-S biogenesis may provide insights in understanding the key Fe-S metabolism for leishmanial survival inside the host macrophage (Fig. 2).

## 2 Experimental design, materials and methods

### 2.1 Homology modeling and model validation

**Fig. 3:**
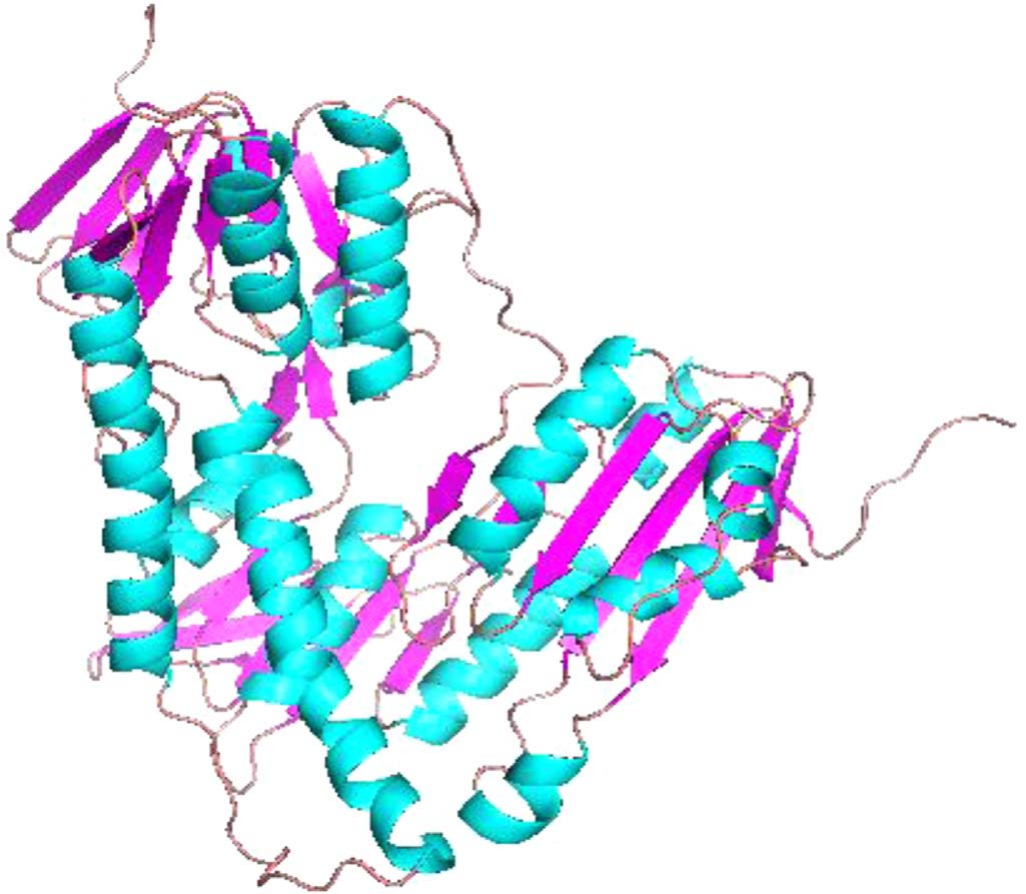
Model of TryR of *L.major*.

The amino acid sequence of the target Trypanothione reductase for *L.major* was obtained from the (National Centre for Biotechnology Information, NCBI) database. For template identification Protein-Protein BLAST (Blastp) was performed for template selection by searching against the Protein Data Bank proteins. One of the homolog structure that had the best score was selected as a template protein. Template protein PDB file and amino acid sequence in FASTA format was downloaded from the Protein Data Bank. A Homology model of TryR enzyme was constructed by using Modeller 9.18. Model was built according to the target sequence, an alignment file and 3D structure of the template protein that was obtained from the Protein Data Bank (PDB).The PDB file of resultant model was visualized by Pymol viewer (Fig. 3).

**Fig. 4:**
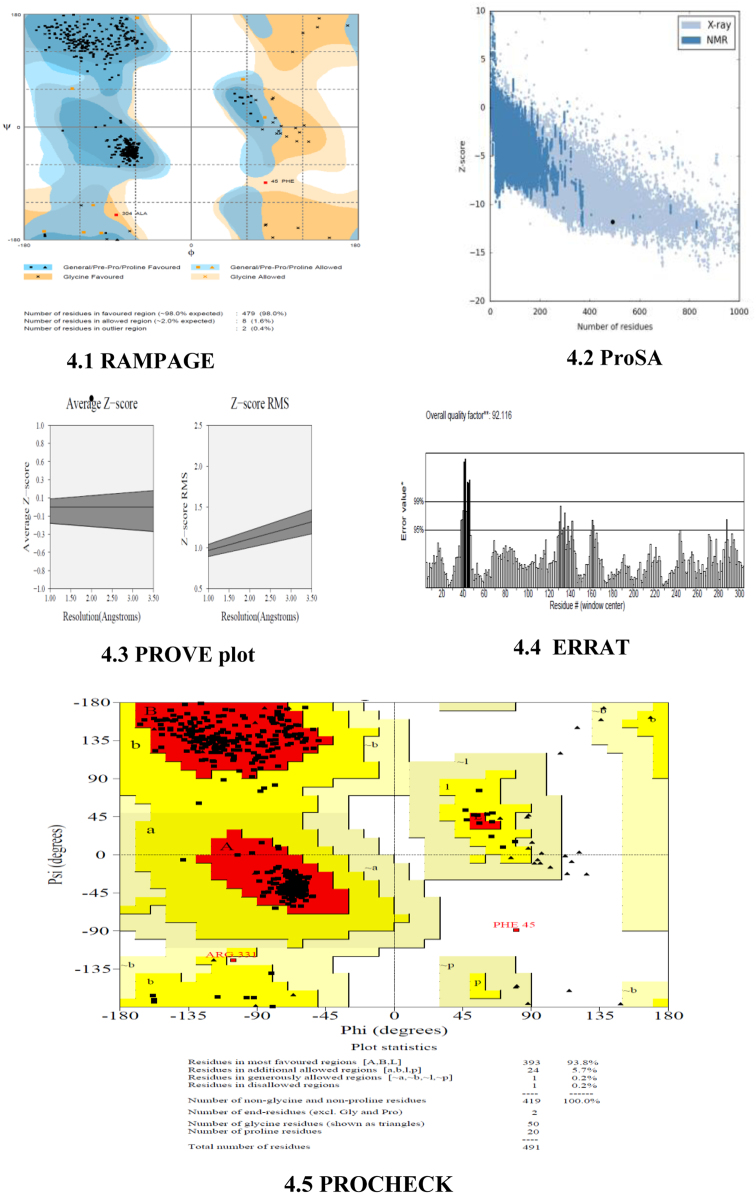
Structure Validation.

Stereochemistry of model was checked by RAMPAGE. Structural quality of the protein was analysed by ProSA web. Various other parameters including buried protein, quality factor, three dimensional score, non-bonded interaction were analysed by SAVES (Fig. 4).

### 2.2 Phylogeny and molecular clock analysis

**Fig. 5:**
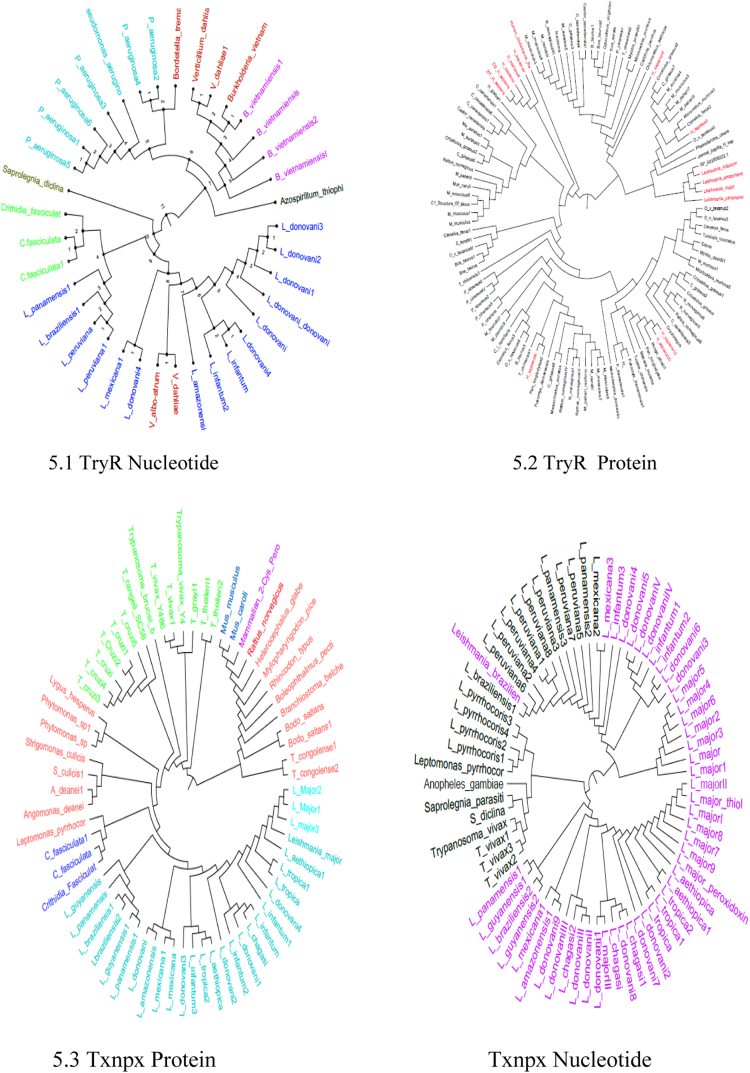
Molecular clock analysis of TryR and Txnpx.

The sequence of TryR (37 Nucleotide sequences and 115 Protein sequences) and Txnpx (72 Nucleotide sequences and 64 Protein sequences) from all organisms ranging from plants to mammals has been taken from NCBI. The sequences obtained from NCBI have been saved in the FASTA format. The sequence has been aligned [17], [18] in a way that it should not possess any partial and hypothetical sequences. These sequences have been given for Multiple Sequence Alignment (MSA) by Clustal Omega in Nexus Format. The Obtained Nexus Format has been given to Mr Bayes 3.2.5 and the resultant tree was viewed using Fig tree v1.4.3exe (Fig. 5).

### 2.3 Molecular docking

**Fig. 6:**
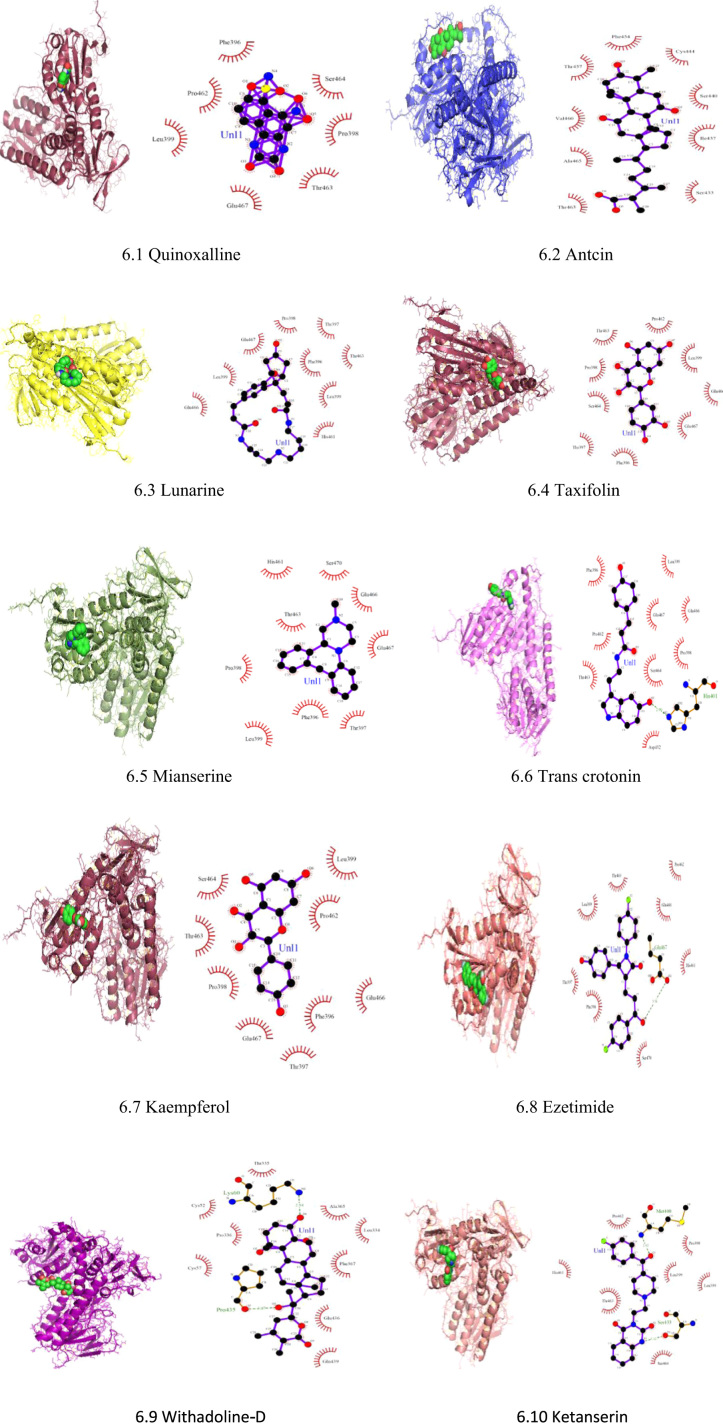
Docking Interaction and LigPlot+ Plot of TryR with various inhibitors.

**Fig. 7:**
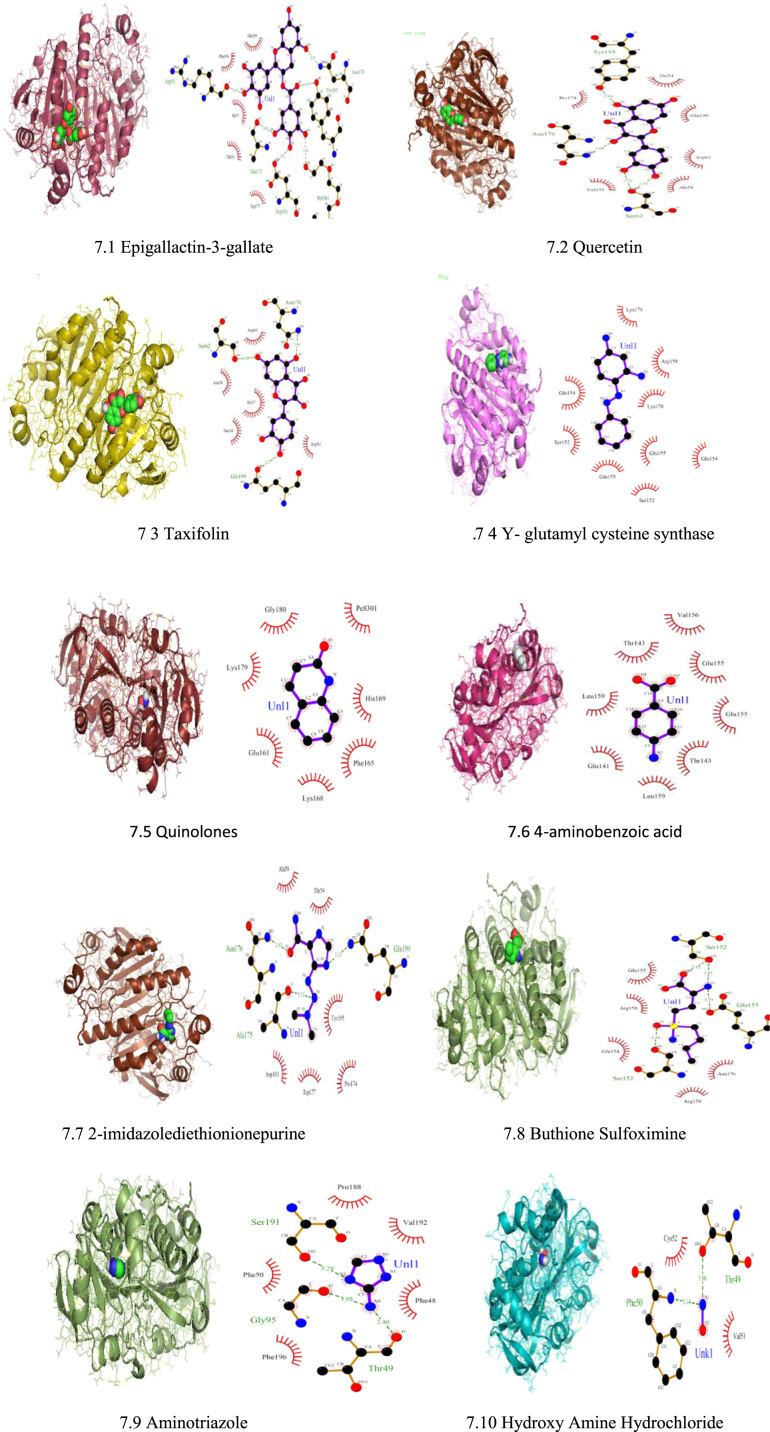
Docking Interaction and LigPlot+ plots of Txnpx with inhibitors.

In docking, the inhibitors were searched against TryR and Txnpx in Leishmania. The compounds were searched using PubChem Bioassay database from NCBI and also through various literature [6], [8], [11], [12], [16], [19]. For TryR, it was found to be 57 inhibitors [Supplementary] and for (Txnpx) it was found to be 17 inhibitors [Supplementary]. These inhibitors were sorted and filtered to 44 inhibitors for TryR and 10 inhibitors for Txnpx by Lipinsky's Rule of Five [12]. The docking has been performed using AutoDock Vina. In AutoDock, the receptor and ligand file has been prepared by deletion of water; addition of polar hydrogen bonds and adding Gasteiger charges to the structure. The grid size has been fixed for all the co-ordinates (x, y and z) to be 50 for TryR and 70 for Txnpx. The docking score obtained depicts the binding of target and inhibitors which describes the best inhibitors with the binding energies expressed as kCal/mol. The docking was analysed by docking score and LigPlot+ to select the best 10 inhibitors for both TryR and Txnpx (Fig. 6, Fig. 7).

### 2.4 Protein network construction

**Fig. 8:**
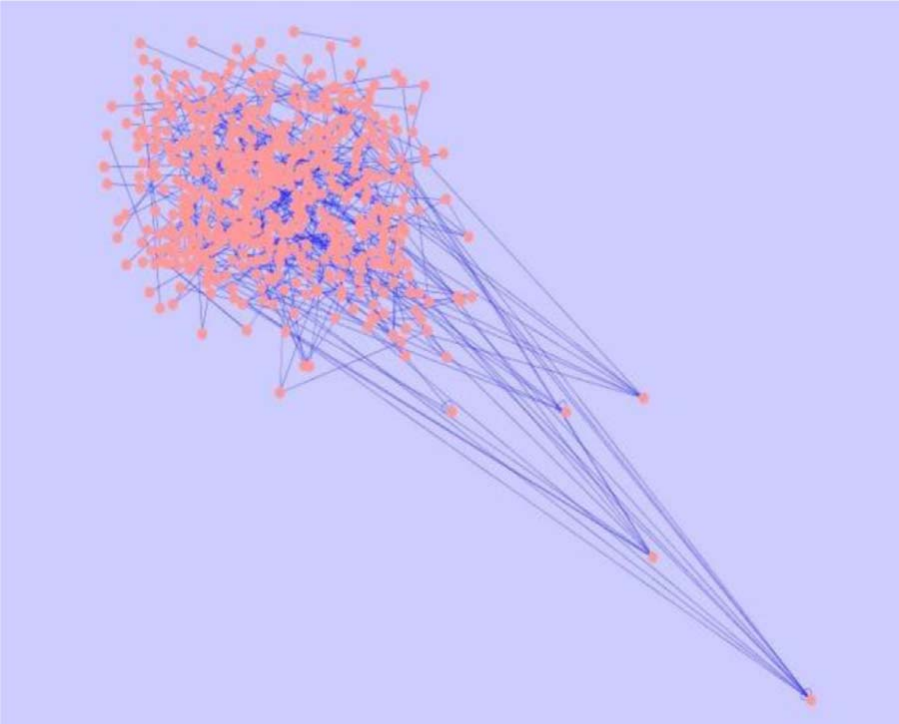
Interaction Network of proteins involved in Redox metabolism of *L.major* after simulated annealing.

To construct the Protein network, the redox metabolism of *L.major* was obtained from KEGG pathway [16]. From the KEGG pathway, the redox metabolism involves 30 Proteins for its survival cycle. To see the neighbouring interaction of those 30 proteins, the various protein interaction databases such as INTACT, STRING, BIND, MINT, HPRD, BIOGRID, MIPS, GRID, DIP were used. From these databases, it was found that, in total, there were 527 interactions for 30 proteins [Supplementary]. The networking of these 527 interactions was constructed and analysed using Cytoscape 3.5.1 [15] (Fig. 8).

## 3 Data processing

### 3.1 Template identification and homology modelling

BLAST analysis was performed for Trypanothione Reductase against Protein Data Bank proteins (PDB). It was found that Chain A structure TryR from *L. infantum* (PDB Id: 2JK6) shared identities of 96% and E-value 0.0. The modeling was done with this template (*L.infantum)* by Modeller 9.18. From modeling, five models have been obtained and the best model was selected for further analysis.

### 3.2 Structure validation

The model obtained from the homology modeling has been validated to find the best three dimensional structures for TryR of *L.major*. The structure was validated using RAMPAGE, ProSA-web and SAVES. In RAMPAGE, it showed that model 2 has low buried region (0.4%) than the other model and 98% of the amino acid residues are in favoured region of the Ramachandran Plot. In ProSA, the Z score of all the models is around -11.7 to -11.9 that indicates the quality of each model. In SAVES, we have various methods for structure validation viz., Prove (to calculate the volume of atoms in macromolecules which describes total number of outlier regions) that showed model 2 has lower outlier region (3.8%); ERRAT (to analyse the statistics of non-bonded interaction describes overall quality factor) showed 92.116% of quality factor for model 2 higher than the other models; Verify 3D (to determine compatibility of an atomic model with its amino acid sequence) showed average score of all the model lies within the range; PROCHECK (to check the stereochemical quality of protein structure). From all this analyses, it was confirmed that model 2 is the best structure for TryR of *L.major* [Supplementary].

After the validation of all the structural models, it is confirmed that the **Model 2** among the five models obtained through homology modelling is the best structural model for TryR of *L.major* and this model may be used further.

### 3.3 Phylogeny and molecular clock analysis

The protein and nucleotide sequence of TryR and Txnpx has been given as an input for Clustal omega against all the organism. The obtained nexus result has been used as an input in Mr Bayes. This result shows that the sequences (not sequencec) of TryR and Txnpx of *L.major* has diverged from the *Homo sapiens* so that these can be used as the potential target for leishmaniasis.

### 3.4 Molecular docking

Initially, 57 Inhibitors for TryR and 17 inhibitors for Txnpx have been sorted out to 44 inhibitors for TryR and 10 inhibitors for Txnpx by Lipinski's rule of five. Then, the docking was performed with the target so that the best inhibitors could be screened through docking score and LigPlot+. This docking has also paved the way to find the binding and active site of the target (Table 1, Table 2).

**Table 1:** Inhibitors of TryR with their respective docking score.

**S.no**	**Inhibitors**	**Docking score**
**1**	Quinoxalline [25]	-10.4
**2**	Antcin [25]	-9
**3**	Lunarine [21]	-8.7
**4**	Taxifolin [11]	-8.3
**5**	Mianserine [20]	-8.3
**6**	Trans crotonin [19]	-8.1
**7**	Kaempferol [20]	-8.1
**8**	Ezetimide [22]	-8.1
**9**	Withanoli-D [19]	-8
**10**	Ketanserin [19]	-7.6

**Table 2:** Inhibitors for Txnpx and its docking score.

**S.no**	**Inhibitors**	**Docking score**
**1**	Epigalloctechin-3-gallate [23]	-7.9
**2**	Quercetin [12]	-7.2
**3**	Taxifolin [12]	-7
**4**	Y-glutamylcysteinsynthatase [23]	-6.1
**5**	Quinolones [24]	-6
**6**	4-aminobenzoic acid [23]	-5.5
**7**	2-imidazoledinethione purin [23]	-5.2
**8**	Butathione Sulfoximine [24]	-4.6
**9**	Aminotriazole [24]	-4.5
**10**	Hydroxy amine hydrochloride [24]	-3

The binding site of TryR in which the inhibitors bound are **Thr463, Glu467, His461, Glu466** and for Txnpx it is found to be **Asn176, Thr54, Ala175, Tyr195**.

### 3.5 Protein-Protein interaction

#### 3.5.1 Network construction

The interaction of protein in the redox metabolism of the leishmania has been noted. The redox pathway of leishmania has been taken in which 30 proteins have been found from KEGG pathway and 527 protein -protein interaction from the other interaction database. Network was constructed by Cytoscape 3.5.1.

#### 3.5.2 Network analysis

**Fig. 9:**
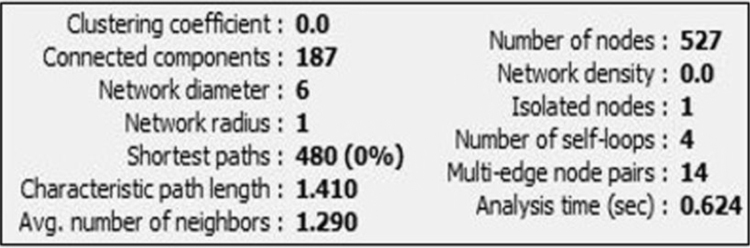
Statistical analysis of simulated annealing network.

After the Network has been constructed, some of the statistical parameters analysed are represented in Fig. 9.

The nodes with less than two neighbours are assumed to have clustering co-efficient of 0 so the network has less than two neighbours; the number of connected components indicates the connectivity of network (strongest or weakest); no edges and solely isolated nodes has a density close to zero; decentralised network will be close to zero; multi edge node pairs indicates neighbouring node linked by more than one edge; the expected distance between the two connected nodes gives characteristic path length. After the simulated annealing, we were able to find that proteins INO1 and RPN5 have the highest connections with maximum clustering coefficient and they are known to be involved in the homeostasis of redox metabolism.

## 4 Data quantification

In *Leishmania* species, reductions of hyper oxidases and reactive oxygen species are provided directly by TryR and Txnpx activities. Because these parasites are sensitive against the oxidative stress, TryR and Txnpx in this pathway are targets for drugs. Reconstruction of phylogenetic tree and its analysis revealed Txnpx has a distant homolog in host species, but it is diverged into another clade, thus, can be a novel target. Similar is the case with Txnpx also. Homology modeling of Trypanothione Reductase protein has given accurate structural model and it was used for further molecular dockings studies. Molecular docking of TryR and Txnpx with different inhibitors from literature was performed and after the analysis, we found a common inhibitor ‘Taxifolin’ for both proteins, is reported to have good affinity against both the proteins. Protein-protein interaction network analysis revealed that the network is sparsely connected and further the statistical analysis indicates that there are certain well studied proteins like INO1 and RPN5 which have a functional interaction and are known to play a vital role in redox metabolism homeostasis.
