# Supplementary material for: Dataset generated for Dissection of mechanisms of Trypanothione Reductase and Tryparedoxin Peroxidase through dynamic network analysis and simulations in leishmaniasis
Source: Data Brief. 2017 Oct 19;15:757–69. doi: 10.1016/j.dib.2017.10.031 (PMC5675996; doi:10.1016/j.dib.2017.10.031)
Supplement: Supplementary file 1 — Supplementary material [file mmc1.docx]

**Conflict of interest**

The authors potentially declares no conflict of interest.
